# Psychometric Properties of the Arabic Version of the Pain Resilience Scale among Lebanese Adults with Chronic Musculoskeletal Pain

**DOI:** 10.1155/2024/7361038

**Published:** 2024-07-29

**Authors:** Melissa Makhoul, Samar Noureddine, Huda Abu-Saad Huijer, Laila Farhood, Souha Fares, Imad Uthman, Douglas J. French, Christopher R. France

**Affiliations:** ^1^ Rafic Hariri School of Nursing American University of Beirut, Beirut, Lebanon; ^2^ Faculty of Health Sciences University of Balamand, Al-Kurah, Balamand, Lebanon; ^3^ Department of Internal Medicine American University of Beirut Medical Center, Beirut, Lebanon; ^4^ Atlantic Pain Clinic, Moncton, New Brunswick, Canada; ^5^ Department of Psychology Ohio University, Athens, OH, USA

## Abstract

**Background:**

The Pain Resilience Scale (PRS), which measures behavioral perseverance and the ability to regulate emotions and cognition despite ongoing pain, lacks an Arabic version.

**Objectives:**

This study aimed to translate, culturally adapt, and validate an Arabic version of the Pain Resilience Scale (PRS-A) among Lebanese adults.

**Methods:**

Phase 1 involved translation and cross-cultural adaptation of the PRS into Arabic. Phase 2 examined the reliability and validity of the PRS-A. A convenience sample of 154 Lebanese adults with chronic musculoskeletal pain completed the PRS-A and self-report measures of pain catastrophizing, pain self-efficacy, pain intensity and interference, depression and anxiety, and quality of life.

**Results:**

The PRS-A yielded a two-factor structure with factor 1 representing “cognitive/affective positivity” and factor 2 representing “behavioral perseverance,” accounting for 41.93% and 15.15% of the variance in pain resilience, respectively. Total PRS-A score (*M* = 33.20 and SD = 9.90) showed significant correlations with pain catastrophizing (*M* = 27.65, SD = 13.03, and *r* = −0.52), pain self-efficacy (median = 9.00, IQR = 4, and rho = 0.61), pain intensity (*M* = 4.50, SD = 2.25, and *r* = −0.28), pain interference (*M* = 4.30, SD = 2.89, and *r* = −0.56), physical (*M* = 34.95, SD = 9.52, and *r* = 0.34) and mental (*M* = 40.08, SD = 12.49, and *r* = 0.58) health functioning, anxiety (median = 7.00, IQR = 7, and rho = −0.57), and depression (median = 4.00, IQR = 6, and rho = −0.58). PRS-A subscale was also significantly related to all measures except pain intensity, which was correlated with cognitive/affective positivity (*r* = −0.33) but not behavioral perseverance (*r* = −0.09). Cronbach's alpha for the PRS-A was 0.87.

**Conclusion:**

The PRS-A demonstrated validity and acceptable reliability among Arab-speaking individuals with chronic musculoskeletal pain, suggesting its potential utility for assessing pain resilience within this population.

## 1. Introduction

Pain of musculoskeletal origin is the most common and disabling form of chronic pain, affecting an estimated 1.71 billion of the population worldwide [1, 2]. The experience of pain is characterized by a combination of physical, psychological, and sociocultural processes, making its treatment challenging [3]. Traditionally, research has focused predominantly on negative psychological factors (e.g., pain catastrophizing and pain-related fear) related to the maintenance and exacerbation of pain [4, 5]. More recently, there has been a paradigm shift towards exploring positive psychological factors that contribute to resilience and promote optimal functioning [6]. Resilience is broadly defined as a dynamic process resulting from the ability to adjust to challenges and maintain successful functioning in the face of adversity [6]. Considerable research has been dedicated to identify factors that contribute to resilience in the context of chronic pain. In light with this, a number of factors (e.g., pain self-efficacy, pain acceptance, optimism, hope, and positive affect) have been identified as protective against chronic musculoskeletal pain and its negative consequences [7–9].

Several measures have been put forward to assess resilience as a general dispositional construct [10, 11]. These measures typically focus on evaluating an individual's belief to overcome adversities. For example, the Brief Resilience Scale (BRS) [10] measures an individual's ability to bounce back from stress, with items such as “I tend to bounce back quickly after hard times.” In addition, other measures of general resilience, such as the Connor–Davidson Resilience Scale (CD-RISC) [11], include items similar to the BRS while also evaluating other constructs related to resilience such as belief about personal competence, spirituality, and close and secure relationships. These generic measures of resilience have been subsequently well-validated and used in previous studies of chronic pain [12, 13]; however, they are less relevant pain than a pain-specific resilience scale as they fail to include resilience elements specifically related to pain, such as positive affect, hope, and optimism [14].

The Pain Resilience Scale (PRS) is the first instrument developed in English by Slepian et al. [14] to measure pain-specific resilience. The PRS captures both the individual's belief in their capacity for resilience, which is a hallmark of general resilience measures, as well as elements of resilience resources specifically related to pain, mainly hope, positive affect, and optimism [14]. The PRS was originally validated in a sample of undergraduate university students with experimental pain. Factor analyses supported a two-factor structure including *behavioral perseverance*, which focuses on behavioral and motivational persistence when confronted with severe or prolonged pain (e.g., “I push through it”), and *cognitive/affective positivity*, which focuses on the perceived ability to regulate emotions and cognition (e.g., “I still find joy in my life”). In addition, the PRS demonstrated good construct validity evidenced by its significant positive correlations with general measures of resilience, and resilience-related constructs (such as pain self-efficacy and hope), as well as negative correlations with vulnerability measures (such as pain catastrophizing, pain-related fear, and pain anxiety) [14]. The PRS was also found to be psychometrically sound in individuals with chronic pain [15], with factor analysis replicating the two-factor structure of the original PRS [14] with two fewer items on the *behavioral perseverance* subscale. Most importantly, the PRS was found to be a stronger predictor of pain-related outcomes than general measures of resilience (e.g., CD-RISC) [14, 15].

More recently, the PRS has been translated into Chinese and Turkish and exhibited good psychometric properties in individuals with chronic musculoskeletal pain [16–18]. Although the PRS has been translated and validated in Chinese [16, 17] and Turkish [18] languages, there is currently no translated and validated version of the PRS for Arabic-speaking populations. Pain-related beliefs (e.g., belief about the cause of pain), appraisals, and coping responses may vary across different countries, language groups, religious beliefs, and economic status [19, 20]. For example, in Arabic cultures, women are expected to manage household chores which may lead some to believe that their pain is the result of their lifestyle. This perception may lead to a decrease in seeking pain treatment, as pain may be considered a lower priority than fulfilling household tasks [21]. Moreover, in Lebanon, pain management remains inadequate and lags behind that of developed countries [22, 23], with many individuals relying on primary healthcare providers for pain treatment, who may be less trained to effectively manage pain [24]. Lebanon has been grappling with the worst economic crisis in its modern history, which has been further exacerbated by the dual economic impact of the COVID-19 outbreak and the massive blast at Beirut's port. These crises have strained the country's healthcare system and exacerbated existing challenges in pain management, with many facing financial barriers and lacking health insurance coverage for essential pain medications and therapies [25, 26]. This situation has also taken a psychological toll on individuals with chronic musculoskeletal pain with many experiencing feelings of hopelessness and fear of disease progression, increased consumption of antidepressants, sleeping pills, anxiolytics, and smoking [26].

Taken together, considering the influence of cultural differences on the pain experience and the unique challenges faced by Lebanese individuals with chronic musculoskeletal pain, the PRS developed by researchers in one culture may not necessarily be understood, appropriate, or effective in the Lebanese culture. Therefore, given the absence of a validated Arabic version of the PRS, the aim of this study was to translate, culturally adapt, and validate the Arabic version of the PRS (PRS-A) in Lebanese adults with chronic musculoskeletal pain. Because the original 14-item version of the PRS was developed and validated in an undergraduate sample with experimental pain [14], we decided to evaluate the psychometric properties of the 12-item version of the PRS previously validated in chronic pain samples [15]. Based on studies of U.S. samples [14, 15] and validation studies in Chinese and Turkish languages [16–18], we hypothesized that the PRS-A would be positively associated with pain self-efficacy and quality of life and negatively associated with pain catastrophizing, anxiety, depression, pain intensity, and pain interference. The availability of a validated PRS-Arabic version will provide valuable insights into how Lebanese adults with chronic musculoskeletal pain are adapting to pain amidst the various socioeconomic and healthcare challenges in the country. It may also assist clinicians and researchers in identifying individuals who may be in most need of resilience interventions to improve adaptation to chronic musculoskeletal pain in Lebanon and other Arab-speaking populations.

## 2. Materials and Methods

The present data were derived from a cross-sectional correlational study, originally designed to examine pain catastrophizing and pain resilience as predictors of patient-reported outcomes in individuals with chronic musculoskeletal pain. This paper reports the first phase of the study, which included the translation and cross-cultural adaptation of the Pain Resilience Scale into the Arabic language (PRS-A) following the guidelines proposed by Beaton et al. [27, 28]. In addition, the researchers tested the psychometric properties of the PRS-A, including internal consistency and construct validity, on 154 native Arabic-speaking adults with chronic musculoskeletal pain.

### 2.1. Phase I

#### 2.1.1. Translation and Cross-Cultural Adaptation

**Figure 1:**
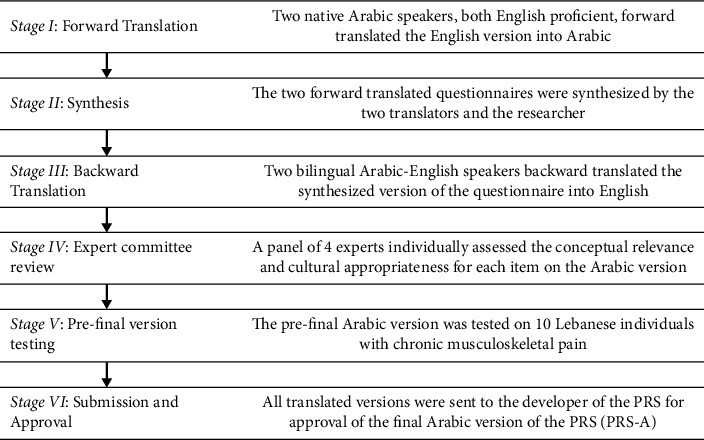
Process of the translation and cross-cultural adaptation of the Pain Resilience Scale (PRS).

Permission was first obtained from the developer of the PRS to translate the tool into Arabic. The PRS was translated using the forward and backward translation method [28]. As displayed in Figure 1, cross-cultural adaptation occurred through five stages. In stage I, following recommendation by Beaton et al. [28] for translator selection, two native Arabic speakers with English proficiency and different profiles independently translated the English questionnaires into Arabic. One translator was a PhD student in Nursing Science who was selected for his clear understanding of the concept being examined with the aim to provide equivalency from a more clinical perspective. The other translator was a professional translator with no medical or clinical background who was selected to ensure linguistic accuracy and cultural appropriateness.

In stage II, these two forward translations were synthesized by the two translators and the researcher. Working from the original English version of the PRS as well as the first translator's (T1) and the second translator's (T2) versions, a comparison was made between the forward and backward translations of the PRS to identify any discrepancies or inconsistencies. These discrepancies were carefully discussed and reviewed, and decisions were made based on the criteria of linguistic accuracy (e.g., ensuring correct grammar and terminology), semantic equivalence (e.g., ensuring the maintenance of the same meaning as the original items), and cultural equivalence (e.g., ensuring that the translated items were relevant and culturally appropriate). For example, the item “I push through it” required careful consideration as it is not a commonly used or culturally relevant sentence in Lebanon. To address this issue, we involved Arabic words and expressions that conveyed a similar meaning of behavioral perseverance. After all discrepancies were resolved, consensus was reached producing one common Arabic version of the PRS (PRS-A).

In stage III, two bilingual Arabic-English speakers independently translated the questionnaire back into the original English language. These translators were selected for their residence in the United States with English being their mother tongue. Most importantly, they were neither aware nor informed about the concepts being explored in the study, thereby ensuring an unbiased back-translation of the PRS-A. The original and back translated versions of the PRS were then compared by the study's research team. All discrepancies were discussed and resolved by consensus.

In stage IV, the PRS-A was tested for content and cultural validity by an expert committee, consisting of one clinical nurse pain specialist, one anesthesiologist and pain and palliative care specialist, one rheumatologist, and one clinical associate professor in Nursing with experience in pain research. The selection of these experts was based on their professional background and experience in the pain field. Guidelines were provided to the expert committee for rating the items of the PRS-A in terms of conceptual relevance and cultural appropriateness. These experts were asked to rate each survey item in terms of its conceptual relevance to the construct and cultural appropriateness to the Lebanese population on a scale of 1 (not relevant/not culturally appropriate) to 4 (highly relevant/very culturally appropriate). Experts were also asked to indicate whether any wording should be revised and to add any comments, if needed. Subsequently, content and cultural validity indices at the scale level (S-CVI) and item level (I-CVI) were calculated [29, 30]. The I-CVI was computed as the number of experts giving a rating of either 3 or 4 (quite relevant/appropriate or very relevant/appropriate), divided by the total number of experts. The S-CVI was calculated by summing the I-CVIs and dividing them by the number of items [29]. An S-CVI of 0.80 or higher and an I-CVI of 0.78 or higher were considered acceptable [29]. As a result, the prefinal PRS-A was established and compared with the original English version to ensure semantic, idiomatic, experiential, and conceptual equivalences.

Lastly, in stage V, the instrument was tested in a pilot study on 10 Lebanese individuals, who were recruited from the outpatient rheumatology clinics of a major referral center, with chronic musculoskeletal pain. Participants' ages ranged from 29 to 64 years, with a mean age of 42.90 years (SD = 12.64): half were female, half were male, and most were married (70%), had completed university education (80%), and were employed full-time (80%). The majority had been experiencing pain for over a year (90%) and had no other comorbidities (60%). Clarity and relevance of the items were checked by asking the participants if any difficulties were encountered while completing the questionnaire. Participants were also asked about the length of the survey and if they had any comments. All translated versions were submitted to the PRS developer who approved the procedure and the final Arabic version of the tool.

### 2.2. Phase II

#### 2.2.1. Participants and Procedure

The sample consisted of 154 individuals (mean age = 43.26; SD = 12.93; range = 18–64 years) with chronic musculoskeletal pain who were recruited from outpatient rheumatology clinics of two medical centers. Participants were included if they were between 18 and 64 years old, had been diagnosed by their physician with chronic musculoskeletal pain, and were able to provide a written informed consent. Participants with chronic musculoskeletal pain were invited to participate by the nurse or the treating physician during their routine follow-up visits. Participants, who met the study eligibility criteria and were interested to know more about the study, were introduced by the nurse or physician to the researcher who informed them about the purpose of the study. Those who agreed to participate following explanation signed the consent form. Afterwards, the researcher administered the questionnaires verbally using a structured interview on-site at the outpatient clinic in a quiet room to ensure privacy.

Participants were excluded if they had (1) chronic nonmusculoskeletal pain based on the ICD-11 pain classifications [31], (2) chronic secondary musculoskeletal pain after musculoskeletal trauma, (3) phantom pain following amputation, (4) present or past DSM-5 diagnosis of schizophrenia, delusional disorder, psychotic disorder, or dissociative disorder, and (5) dementia. Ethical approval for the original study was provided by the Institutional Review Board and Ethics Committee in the University and recruitment sites where the study was conducted. All participants were informed about the study purpose and procedure and signed a written informed consent prior to study enrolment. Participants did not receive compensation or incentives for their involvement in the study.

#### 2.2.2. Sample Size

A priori sample size was calculated for the parent study using G^*∗*^Power software based on 14 predictors, a power of 80%, a probability of alpha error of 0.01, and an effect size of 0.20, based on a study where pain resilience and pain catastrophizing jointly predicted 17% of the variance in mental health-related quality of life [32]. In validation studies, a participant-to-item ratio of 10 : 1 is recommended (10 participants per item on the PRS) [33]. Thus, a sample size of 154 is adequate to test the psychometric properties of the 12-item PRS.

#### 2.2.3. Measures

The study questionnaire included the PRS-A, pain self-efficacy questionnaire (PSEQ), pain catastrophizing scale (PCS), Hospital Anxiety and Depression Scale (HADS), Short Form Brief Pain Inventory (SF-BPI), and Short Form Health Survey-Arabic Version (SF-12), in addition to demographic and clinical questions.

#### 2.2.4. Pain Resilience Scale-Arabic Version (PRS-A)

Given that our sample consisted of adults with chronic musculoskeletal pain and the original 14-item version of the PRS was validated on an undergraduate sample with experimental pain [14], we used the 12-item version that was validated in U.S adults who had chronic pain [15]. The PRS-A is an Arabic-language translation of the 12-item PRS, which contains five items on behavioral perseverance (e.g., “I try to continue working”) and seven items on cognitive/affective positivity (e.g., “I keep a positive attitude”). Items are scored using a 4-point Likert scale (0 = not at all and 4 = all the time) yielding a summative score range from 0 to 56, with higher scores indicating greater pain resilience. The 12-item PRS supported the original two-factor structure [14] and demonstrated adequate reliability and validity when tested in chronic pain samples [15, 17].

#### 2.2.5. Pain Self-Efficacy Questionnaire-Arabic Version (PSEQ-A)

The original version of the PSEQ-A consists of 10 items based on adults with chronic low back pain which demonstrated good psychometric properties [34]. We used the 2-item short form version (PSEQ-2) based on a U.S study of adults with chronic pain to decrease participant burden [35]. The PSEQ-2 assesses the confidence in one's ability to engage in daily activities despite their pain (e.g., “I can live a normal lifestyle, despite the pain”). Items are scored using a 6-point Likert scale (0 = not at all confident and 6 = completely confident), yielding a summative score range from 0 to 12, with higher scores indicating greater pain self-efficacy. The PSEQ-2 has shown adequate reliability and validity, and its psychometric properties were comparable to those of the original 10-item PSEQ [36]. In this study, the two items had a significant and positive correlation (rho = 0.60).

#### 2.2.6. Pain Catastrophizing Scale-Arabic Version (PCS-A)

The PCS-A is the translated Arabic version [37] of the original PCS [38], which consists of 13 items that assess different thoughts and beliefs about the pain experience. The PCS-A contains four items on *rumination* (e.g., “I keep thinking about how much it hurts”), three items on *magnification* (e.g., “I wonder whether something serious may happen”), and six items on *helpfulness* (e.g., “I feel I can't stand it anymore”). Items are scored using a 5-point Likert scale (0 = not at all and 4 = all the time), yielding a summative score range from 0 to 52, with higher scores indicating greater pain catastrophizing. Huijer et al. [28] showed that the Arabic version of the PCS demonstrated adequate psychometric properties comparable to the original version [38] in a sample of Lebanese adults with chronic nonmalignant pain. In this sample, Cronbach's *α* for PCS-A was 0.89.

#### 2.2.7. Hospital Anxiety and Depression Scale-Arabic Version (HADS-A)

The HADS-A is the translated Arabic version [39] of the original HADS [40] and consists of 14 items that measure symptoms of depression and anxiety in nonpsychiatric individuals. The HADS-A contains seven items for the *anxiety subscale* (HADS-A) and seven for the *depression subscale* (HADS-D). Items are scored using a 4-point Likert scale with scores ranging between 0 and 3. Summative scores are calculated for each subscale, with minimum scores of 0 and maximum scores of 21 for each. The HADS-A showed good reliability and validity among individuals admitted for surgical procedure in Saudi Arabia [41] and was also found to be reliable in Lebanese individuals on dialysis [42]. In the present study, Cronbach's alpha coefficients for the HADS-A and the HADS-D subscales were 0.81 and 0.80, respectively.

#### 2.2.8. Short Form Brief Pain Inventory-Arabic Version (SF-BPI-A)

The SF-BPI-A is the translated short form Arabic version [43] of the original BPI [44], which consists of four items that measure *pain intensity* and seven items that measure *pain interference*. Each item is rated from 0 = no pain/no interference to 10 = worst possible pain/pain totally interferes. Average summative scores are calculated for each subscale, with a minimum score of 0 and maximum of 10 for each. Ballout et al. [34] replicated the two-factor structure for *pain severity* and *pain interference* and demonstrated adequate reliability and validity of the short form BPI-A in a sample of Lebanese oncology individuals with chronic pain. Cronbach's alphas for the BPI Pain Intensity subscale and the BPI Pain Interference subscale in the present study were 0.81 and 0.89, respectively.

#### 2.2.9. Short Form Health Survey-Arabic Version (SF-12-A)

The SF-BPI-A is the translated short form Arabic version [45] of the original SF-12 [46], which consists of six items that measure *physical health functioning* and six other items that measure *mental health functioning*. The physical and mental health subscales total scores are computed using the scores of the 12 items, ranging from 0 to 100, where 0 indicates the lowest level of health measured and 100 indicates the highest level of health. The scores are converted to the United States norm-based scoring algorithm, which employs a T-score transformation with a mean of 50 and standard deviation of 10 [47]. Data on the psychometric properties of the SF-12 Arabic version in a sample of Lebanese healthy adults with physical or mental illnesses yielded a two-factor structure and adequate reliability and validity [45]. In this study, Cronbach's alpha for the physical health functioning and mental health functioning subscales were 0.79 and 0.77, respectively.

#### 2.2.10. Statistical Analysis

All data were analysed using the Statistical Product and Service Solution (SPSS) version 26.0. Descriptive statistics were used to summarize participants' clinical and sociodemographic characteristics. Exploratory factor analysis (EFA) was conducted to examine the factor structure of the PRS-A in Lebanese adults with chronic musculoskeletal pain. We opted for EFA because this study is the first to translate and validate the PRS in the Arab world. Evidence suggests that pain perception may be influenced by cultural differences, linguistic variations, disparities in economic status, and healthcare access [20, 48]. Given the linguistic and cultural differences between Lebanon and regions where the PRS has been previously validated, such as the United States, China, and Turkey, it is essential to consider potential differences in pain perception. In addition, the limited access to pain management services, economic challenges, and increased stress experienced by Lebanese adults with chronic musculoskeletal pain [24–26] underscore the importance of examining whether the Arabic adaption of the PRS would yield a factor structure that is comparable with the original English language version. A principle component analysis (PCA) with ProMax rotation was used. The Kaiser–Meyer–Olkin (KMO) measure of sampling adequacy and Bartlett's test of sphericity were calculated. KMO values between 0.80 and 1.0 were considered adequate, and a significant Bartlett test shows that the data are appropriate for factor analysis [49]. The number of factors to be extracted was required to have an eigenvalue greater than 1.0 [50].

Construct validity was also examined by correlating (Pearson's or Spearman's rho) the PRS-A with other positive psychological measures (PSEQ), vulnerability measures (PCS, HADS-A, and HADS-D), and pain-related outcomes including pain intensity and pain interference (BPI), and health-related quality of life scores (SF-12). Correlational analysis was selected as it provides valuable insights into construct validity of the PRS-A by examining its relationships with other theoretically related variables [51].

Cronbach's alpha coefficient (*α*), corrected item-total correlations, Cronbach's alpha if item deleted, and average interitem correlation were used to examine the internal consistency of the PRS-A. Finally, floor and ceiling effects were examined by determining the proportion of participants who had the minimum and maximum scores on the PRS-A and considered to exist if >15% of the participants had minimum and maximum scores on the PRS-A [52].

## 3. Results

### 3.1. Translation and Cross-Cultural Adaptation

No problems were encountered during the translation and cross-cultural adaptation steps. Specific criteria were used for the evaluation of the translated questionnaire by the expert committee, which included aspects related to conceptual relevance, linguistic clarity, cultural appropriateness, and overall comprehensibility of the items. The majority of the PRS-A items were rated by the expert committee at 3 or 4 (quite relevant/appropriate or highly relevant/appropriate) on conceptual relevance and cultural appropriateness. The conceptual relevance and cultural appropriateness ratings for each item are provided as electronic Supplementary Materials (Tables 1 and 2, respectively). The scale content validity index S-CVI/Ave was 0.88 and the cultural validity index S-CVI/Ave was 0.96. For content validity at the item level, 50% of the I-CVI ratings were greater than 0.78 and the other 50% were at 0.75. On the other hand, for cultural validity, 83% of I-CVI ratings were greater than 0.78 and only 17% were at 0.75. None of the items had ratings below 0.50, which would indicate rejection of the item. Disagreements among experts were resolved through discussion and reaching consensus. For example, item 5 “I like to stay active” received a score of 2 out of 4 from one expert in the field. The reason for this score was that in the Arabic language, the translation of “active” can have multiple interpretations, including being physically active or being active in multiple areas (physically, socially, and mentally active). To ensure clarity and accuracy, this term was modified to signify being active not only physically but also in other aspects such as social or mental activity. Furthermore, during pilot testing, participants indicated that all items on the PRS-A were clear, relevant, and easy to understand and rate. In addition, no concerns were raised about the questionnaire's length. The final version of the PRS-A was approved by the original PRS developer (Table 1 shows the English version of the PRS).

### 3.2. Psychometric Properties of the PRS-A

#### 3.2.1. Sample Characteristics

Participants' ages ranged from 18 to 64 years. The majority were females, had a university education, were currently married, were unemployed, and had an annual household income of ≤$6,000. Most of the participants had been experiencing chronic musculoskeletal pain for more than a year with no other comorbidities. According to the International Classification of Diseases (ICD)-11of chronic pain [31, 53], 60.4% of participants were diagnosed with chronic secondary musculoskeletal pain (CSMP), 20.8% with chronic primary musculoskeletal pain (CPMP), and 18.8% experienced both CPMP and CSMP. Participants' total mean score on the PRS-A was 33.20 (SD = 9.90 and range: 9–48). More sample characteristics and descriptive statistics of study measures are reported in Table 2.

#### 3.2.2. Construct Validity

There were no missing data in any of the variables. The KMO coefficient was 0.855, indicating that the sample size was sufficient. The Bartlett test of sphericity was statistically significant (*p* < 0.001). These results showed that the data distribution was appropriate for factor analysis. According to the EFA result, the PRS-A had a two-factor structure with eigenvalues greater than 1, explaining 57.08% of the variance. The communalities for each item were all above 0.4 except for item 5 (I like to stay active) with only 26.7% of its variance explained by the factors. The first component included seven items for cognitive/affective positivity and explained 41.93% of the scale variance. The second component comprised all five behavioral perseverance items and accounted for 15.15% of the scale variance. All items met the minimum criterion of having a primary factor loading of 0.4 or above and no item loaded on more than one factor. Item loadings and communalities are presented in Table 3.

Further testing for construct validity was done by examining correlations between the PRS-A scores and obtained scores on measures known to relate to resilience in specific ways, namely, pain self-efficacy, pain catastrophizing, anxiety and depression, pain intensity and interference, and quality of life. The scores of all questionnaires were normally distributed, except for the PRS-behavioral perseverance subscale scores, PSEQ scores, HADS-A, and HADS-D scores, so nonparametric correlation analyses were used accordingly. As displayed in Table 4, the PRS-A scores demonstrated significant moderate positive associations with pain self-efficacy and mental health functioning, and significant weak positive association with physical health functioning (all *p* < 0.001). Furthermore, the PRS-A demonstrated significant moderate negative associations with pain catastrophizing, anxiety, depression, and pain interference, and significant weak negative association with pain intensity (all *p* < 0.001). Correlations of the PRS subscale scores with pain self-efficacy, pain catastrophizing, anxiety, depression, pain interference, and physical and mental health functioning were also significant (all *p* < 0.001). As for pain intensity, it was significantly and negatively associated with the cognitive/affective positivity subscale (*p* < 0.001) but not with the behavioral perseverance subscale (*p* = 0.126), as shown in Table 4.

#### 3.2.3. Internal Consistency

A reliability analysis was conducted on the PRS-A. All 12 items of the PRS were measured on a 4-point Likert scale and worded in the same direction. Cronbach's alpha coefficient was *α* = 0.87. All items correlated well together (*r* > 0.3), except for item 12 “I try to stay relaxed” (*r* = 0.23). The average interitem correlation was 0.71. Only item 12 showed a minor increase in the alpha if deleted (up to 0.88). Results for reliability analysis of the total PRS-A are displayed in Table 5. For the subscales, Cronbach's alpha coefficients for the scores of the PRS-cognitive/affective positivity and PRS-behavioral perseverance subscales were 0.85 and 0.81, respectively. Cronbach's alpha if item deleted of PRS ranged from 0.811 to 0.862 for PRS cognitive/affective positivity, and from 0.722 to 0.831 for PRS-behavioral perseverance.

#### 3.2.4. Floor and Ceiling Effects

Floor and ceiling effects were assessed for the PRS-A and its subscales. None of the participants had a total score of 0 on the PRS-A, while seven participants (4.55% of the sample) had a score of 48; thus, no floor and ceiling effects were observed. On the cognitive/affective positivity subscale, none of the participants had a score of 0 on the cognitive/affective positivity and seven participants (4.55% of the sample) had a score of 28, indicating no floor and ceiling effects. Similarly, on the behavioral perseverance subscale, none of participants had a score of 0; however, thirty-eight (24.7% of the sample) had a score of 20 suggesting the presence of a potential ceiling effect.

## 4. Discussion

The present study aimed to translate, culturally adapt, and evaluate the psychometric properties of the PRS-A in Lebanese adults with chronic musculoskeletal pain. Study findings revealed that the PRS-A is understandable, reliable, and valid, suggesting its usefulness for clinical use among Arab health professionals for adults with chronic musculoskeletal pain.

### 4.1. Factor Structure of the PRS-A

The present data revealed a 12-item, two-factor PRS structure, fully replicating the original cognitive and behavioral perseverance subscales [14, 15]. The first component, “cognitive/affective positivity,” reflects a person's ability to still find joy in life despite the pain. This is consistent with theoretical frameworks of resilience, which holds that positive psychological resources such as optimism and hope help build resilience and optimal functioning. The second component, “behavioral perseverance,” reflects a person's ability to continue working or performing activities of daily living despite the pain. This aligns with the emphasis on “approach coping” for resilience rather than “avoidance coping,” which may help mitigate negative pain-related outcomes [54]. The same pattern of EFA results was found in the previous study that validated the 12-item PRS in Chinese language among adults with chronic musculoskeletal pain, but with two fewer items that failed to load on the “*behavioral perseverance*” factor [16]. In our sample, all twelve items had factor loadings that exceeded the threshold (above 0.30 or 0.40) recommended by Floyd and Widaman [55], suggesting that items in the PRS-A are reflections of the same underlying construct. Particularly, similar item groupings to the original 2-factor PRS model were found, where all seven items aligned with the cognitive/affective construct and all five items with behavioral perseverance factor. To conclude, the replication of the two-factor structure of the PRS enhances cross-cultural validity, as it indicates that the scale's items are understood similarly, which is critical for ensuring the scale's applicability across cultures. Accordingly, users of the 12-item PRS-A can feel comfortable scoring this measure based on Ankawi et al.'s [15] original 2-factor item groupings and when using the total score of the instrument.

### 4.2. Construct Validity and Internal Consistency

As hypothesized, results of this study demonstrated that higher scores on the PRS-A were associated with significantly higher levels of pain self-efficacy and physical and mental health functioning and lower levels of pain catastrophizing, anxiety, depression, pain intensity, and interference. These results are consistent with the English [14, 15], Chinese [16, 17], and Turkish [18] versions of the PRS, all of which support the importance of targeting both vulnerability and resilience factors in chronic pain. Increasing pain resilience is likely to lead to decreased disability and lower levels of depression and anxiety. This latter point is important given the high co-occurrence of chronic pain and depression and/or anxiety [56]. These observed associations between the PRS-A and pain-related outcomes suggest the importance of incorporating assessments of pain resilience into chronic pain management practices to better understand the person's pain experiences and tailor interventions accordingly. Future research studies may focus on developing resilience-based interventions tailored to improve pain resilience and overall well-being within Arab populations.

The PRS-A also exhibited good internal consistency, with Cronbach's alpha coefficients being in a similar range or slightly lower than values obtained in the original English study (0.87 to 0.93) [14], the Chinese validation study (0.89 to 0.92) [16], and Turkish validation study (0.86 to 0.92) [18], yet above 0.70. The average interitem correlation was 0.71, suggesting that the items in the scale are highly correlated with each other which help ensure the homogeneity of the scale. However, it is important to note that values above 0.50 may also indicate item redundancy [57]. Therefore, while these results suggest that the PRS-A is a reliable tool, it may be beneficial for future studies to consider revising the items to improve the scale's accuracy.

### 4.3. Floor and Ceiling Effects

Results of this study also showed that there were no significant floor and ceiling effects for the total PRS-A score and its cognitive affective subscale, which is comparable to the Turkish version of the PRS [18]. However, a ceiling effect was present for the behavioral perseverance subscale, which may have occurred due to social desirability. Specifically, measures assessing positive psychological constructs may be typically associated with social desirability bias due to their association with flourishing [58], making them generally desirable constructs. As such, some participants might have over-reported items on the behavioral perseverance subscale, whether unconsciously to preserve a positive self-image or consciously to produce a more favourable impression to the researcher. Future research studies could use social desirability measures such as the Marlowe–Crowne Social Desirability Scale (MC–SDS) [59] to further examine the relationship between social desirability and the PRS-A. In addition, we highlight the importance of revising and potentially adding new items to capture more nuanced aspects of behavioral perseverance relevant to the population.

The ceiling effect on the behavioral perseverance subscale may also be attributed to the large proportion of female participants in this study (91%). The PRS-B included items such as “I like to stay active,” “I still work to accomplish my goals,” “I push through it,” “I try to continue working.” Participants' responses to these items could have been influenced by the cultural context of women in Lebanon. As in most of the Arabic region, Lebanese women are largely expected to fulfil traditional household responsibilities, including cleaning, cooking, and caring for children [60], which may explain the positive responses on items related to the pursuit and accomplishment of activities despite ongoing pain. This warrants the need for future research to explore the meaning of resilience among adults living with chronic pain in the Arab region, particularly in Lebanon.

### 4.4. Strengths and Limitations

To our knowledge, this is the first study to translate, culturally adapt, and validate the PRS into the Arabic language. An additional strength of this study is that it used a robust translation methodology. The availability of an Arabic version of the PRS may help assist clinicians and researchers in assessing pain resilience and developing or implementing culturally relevant resilience-based interventions tailored to the Arab population. Also, it may facilitate cross-cultural comparisons and collaborative research efforts, enabling researchers to explore pain resilience across different cultures.

Nevertheless, similar to other research studies, this study is not without limitations. Participants in this sample were mostly females (90.9%), which may limit the generalizability of the study findings to males with chronic musculoskeletal pain. Future studies should include more male participants, as research suggests that sex differences may influence pain coping responses, with women being more likely to engage in catastrophic thoughts and expressing pain more openly than men due to societal norms [61]. Another limitation is that we only assessed some measures of reliability and validity. As such, further research is needed to examine other important parameters related to understanding mechanisms of change in treatment such as predictive validity and test-retest reliability. Lastly, our small sample size did not allow us to conduct both EFA and confirmatory factor analysis (CFA), as CFA requires a separate dataset than EFA, which would imply splitting the sample into half [62]. However, the EFA results enabled us to examine a factor structure that proved to be comparable to the original English version, despite the linguistic and cultural differences between Lebanon and the U.S. We recommend that future studies examining the validity of the PRS-A consider conducting CFA to allow for a confirmation of the underlying factor structure of the PRS-A.

## 5. Conclusions

This study provides preliminary evidence of the reliability and validity of the Arabic version of the PRS (PRS-A) in adults with chronic musculoskeletal pain. The resulting two-factor structure fully replicating results of Ankawi et al.'s [12] study of U.S. adults with chronic pain. While further evaluation of the instrument in other clinical settings and in other Arab cultures is needed, we recommend the use of the PRS-A by researchers and clinicians in the assessment of pain-related resilience in Arab individuals with chronic musculoskeletal pain. We hereby authorize others to use this measure in their research or clinical work.

## Figures and Tables

**Table 1:** English version of the Pain Resilience Scale (PRS).

When faced with intense or prolonged pain…		Not at all	To a slight degree	To a moderate degree	To a great degree	All the time
(1)	I get back out there	0	1	2	3	4
(2)	I still work to accomplish my goals	0	1	2	3	4
(3)	I push through it	0	1	2	3	4
(4)	I try to continue working	0	1	2	3	4
(5)	I like to stay active	0	1	2	3	4
(6)	I keep a positive attitude	0	1	2	3	4
(7)	It does not affect my happiness	0	1	2	3	4
(8)	I still find joy in my life	0	1	2	3	4
(9)	I keep a hopeful attitude	0	1	2	3	4
(10)	I do not let it get me down	0	1	2	3	4
(11)	I avoid negative thoughts	0	1	2	3	4
(12)	I try to stay relaxed	0	1	2	3	4

*Note*. We are interested in the different ways that you respond to your intense or prolonged pain. Using the following 0 (not at all) to 4 (all the time) scale, please indicate how much each of the following items describe how you respond when faced with intense or prolonged pain.

**Table 2:** Sample characteristics and descriptive statistics of study measures (*N* = 154).

Variables	*N* (%)	Mean (SD)
Age (years)		43.26 (12.93)
Gender		
Female	140 (90.9)	
Education		
≤Middle school	30 (19.5)	
High school	26 (16.9)	
University	98 (63.6)	
Marital status		
Married	102 (66.2)	
Employment		
Unemployed	86 (55.8)	
Household annual income		
≤$6,000	66 (42.9)	
$6,000–11,999	41 (26.6)	
$12,000–14,999	13 (8.4)	
≥$15,000	34 (22.1)	
Musculoskeletal disorder^1^		
Chronic primary musculoskeletal pain^1^	61 (39.6)	
Fibromyalgia	49 (80.3)	
Cervical pain	2 (3.28)	
Low back pain	9 (14.75)	
Limb pain	2 (3.28)	
Nonspecific joint and muscle pain	2 (3.28)	
Chronic secondary musculoskeletal pain	122 (79.2)	
Inflammation	107 (87.7)	
Due to infection	1 (0.9)	
Crystal deposition (gout)	3 (2.8)	
Autoimmune and autoinflammatory disorders	103 (96.3)	
Structural changes	21 (17.2)	
Osteoarthritis	10 (47.6)	
Spondylosis	14 (66.7)	
Scoliosis	1 (4.76)	
Nervous system	1 (0.82)	
Multiple sclerosis	1 (100)	
Pain duration		
≤12 months	17 (11.0)	
>12 months	137 (89.0)	
Comorbidities		
No comorbidities	84 (54.5)	
One comorbidity	38 (24.7)	
Multimorbidity (2 or more)	32 (20.8)	
Study measures		
PRS		33.20 (9.90)
PCS		27.65 (13.03)
BPI-pain intensity		4.50 (2.25)
BPI-pain interference		4.30 (2.89)
SF-12-PCS		34.95 (9.52)
SF-12-MCS		40.08 (12.49)

		Median (IQR)

PSEQ		9.00 (4)
HADS-depression		4.00 (6)
HADS-anxiety		7.00 (7)

^1^Valid percent more than 100 because 29 (18.8%) participants had both chronic primary and chronic secondary musculoskeletal pain, and some also had multiple pain diagnoses within the same category (e.g., one participant had chronic cervical, limb, and low back pain). PCS: pain catastrophizing scale; PRS: pain resilience scale; PSEQ: pain self-efficacy questionnaire; BPI: brief pain inventory; SF-12-PCS: short-form-12-physical composite score; SF-12-MCS: short form-12 mental composite score; HADS: hospital anxiety and depression scale.

**Table 3:** Exploratory factor analysis results for the Pain Resilience Scale among Lebanese adults with chronic musculoskeletal pain.

	Factor		Communalities
	Factor 1 (cognitive/affective positivity)	Factor 2 (behavioral perseverance)	
Item 1: I get back out there	0.054	**0.733**	0.574
Item 2: I still work to accomplish my goals	0.006	**0.867**	0.756
Item 3: I push through it	−0.042	**0.723**	0.498
Item 4: I try to continue working	−0.070	**0.893**	0.749
Item 5: I like to stay active	0.164	**0.425**	0.267
Item 6: I keep a positive attitude	**0.598**	0.208	0.508
Item 7: It does not affect my happiness	**0.506**	0.283	0.459
Item 8: I still find joy in my life	**0.762**	0.069	0.631
Item 9: I keep a hopeful attitude	**0.743**	0.049	0.586
Item 10: I do not let it get me down	**0.735**	0.157	0.665
Item 11: I avoid negative thoughts	**0.820**	−0.004	0.669
Item 12: I try to stay relaxed	**0.760**	−0.455	0.486
Eigenvalue	5.031	1.818	
Percentage of variance explained	41.929	15.147	

The bold values represent loadings above 0.4, indicating a strong relationship between the variables and their respective factors.

**Table 4:** Convergent validity of the Arabic version of the Pain Resilience Scale and its cognitive/affective positivity and behavioral perseverance subscales.

Variables	PRS-A	PRS-A cognitive/affective positivity	PRS-A-behavioral perseverance
PSEQ	0.61^*∗∗*^^1^	0.51^*∗∗*^^1^	0.56^*∗∗*^^1^
PCS	−0.52^*∗∗*^	−0.53^*∗∗*^	−0.33^*∗∗*^^1^
HADS-A	−0.57^*∗∗*^^1^	−0.61^*∗∗*^^1^	−0.31^*∗∗*^^1^
HADS-D	−0.58^*∗∗*^^1^	−0.60^*∗∗*^^1^	−0.35^*∗∗*^^1^
Pain intensity of the BPI	−0.28^*∗∗*^	−0.33^*∗∗*^	−0.09
Pain interference of the BPI	−0.56^*∗∗*^	−0.55^*∗∗*^	−0.36^*∗∗*^^1^
Physical health functioning of the SF-12	0.34^*∗∗*^	0.21^*∗*^	0.42^*∗∗*^^1^
Mental health functioning of the SF-12	0.58^*∗∗*^	0.63^*∗*^	0.34^*∗∗*^^1^

^1^Spearman's rho was calculated. ^*∗*^Correlation is significant at the 0.05 level (2-tailed). ^*∗∗*^Correlation is significant at the 0.01 level (2-tailed). PRS-A: pain resilience scale Arabic; PSEQ: pain self-efficacy questionnaire; PCS: pain catastrophizing scale; HADS-A: hospital anxiety and depression scale-anxiety subscale; HADS-A: Hospital Anxiety and Depression Scale-Depression subscale; BPI: brief pain inventory; SF-12: short form-12.

**Table 5:** Mean scores and corrected item-total correlation deleted results for the total PRS-A.

Items	Mean	SD	Corrected item-total correlation
Item 1: I get back out there	2.31	1.62	0.55
Item 2: I still work to accomplish my goals	2.97	1.34	0.62
Item 3: I push through it	3.33	1.08	0.47
Item 4: I try to continue working	2.95	1.32	0.58
Item 5: I like to stay active	3.45	0.98	0.40
Item 6: I keep a positive attitude	2.74	1.28	0.61
Item 7: It does not affect my happiness	1.77	1.41	0.58
Item 8: I still find joy in my life	2.90	1.29	0.65
Item 9: I keep a hopeful attitude	2.94	1.29	0.60
Item 10: I do not let it get me down	2.82	1.28	0.70
Item 11: I avoid negative thoughts	2.59	1.25	0.65
Item 12: I try to stay relaxed	2.41	1.29	0.23

## Data Availability

The data used to support the findings of this study are available from the corresponding author upon request.

## Supplementary Materials

Supplementary Materials Supplementary tables are provided and include a detailed description of the conceptual (Table 1) and cultural (Table 2) appropriateness ratings of the Pain Resilience Scale (PRS) by the expert committee.
