# Supplementary material for: Psychometric Properties of the Arabic Version of the Pain Resilience Scale among Lebanese Adults with Chronic Musculoskeletal Pain
Source: Pain Res Manag. 2024 Jul 29;2024:7361038. doi: 10.1155/2024/7361038 (PMC11300090; doi:10.1155/2024/7361038)
Supplement: Supplementary Materials — Supplementary tables are provided and include a detailed description of the conceptual (Table 1) and cultural (Table 2) appropriateness ratings of the Pain Resilience Scale (PRS) by the expert committee. [file 7361038.f1.docx]

**Supplementary Materials**

Supplementary Table 1

*Conceptual relevance of the Arabic version of the Pain Resilience Scale (PRS-A)*

|  | **Conceptual Relevance** | | | |  |  |
| --- | --- | --- | --- | --- | --- | --- |
| Please rate the relevance of each item of the scale below on a 4-point Likert scale (1 = Not at All Relevant to 4 = Highly Relevant) | Expert 1 | Expert 2 | Expert 3 | Expert 4 | Number of Experts who scored 3 or 4 on the item | Item I-CVI |
| 1. I get back out there | ___ | X | X | X | **3** | **0.75** |
| 1. I still work to accomplish my goals | X | X | X | X | **4** | **1.00** |
| 1. I push through it | X | X | X | X | **4** | **1.00** |
| 1. I try to continue working | X | X | X | X | **4** | **1.00** |
| 1. I like to stay active | X | X | ___ | X | **3** | **0.75** |
| 1. I keep a positive attitude | X | X | X | X | **4** | **1.00** |
| 1. It doesn’t affect my happiness | X | X | X | X | **4** | **1.00** |
| 1. I still find joy in my life | X | __ | X | X | **3** | **0.75** |
| 1. I keep a hopeful attitude | X | X | X | X | **4** | **1.00** |
| 1. I don’t let it get me down | X | X | X | ___ | **3** | **0.75** |
| 1. I avoid negative thoughts | X | X | X | ___ | **3** | **0.75** |
| 1. I try to stay relaxed | X | X | X | ___ | **3** | **0.75** |
| S-CVI/Ave | **0.875** | | | | | |

Supplementary Table 2

*Cultural appropriateness of the Arabic version of the pain resilience scale (PRS-A)*

|  | **Cultural Appropriateness** | | | |  |  |
| --- | --- | --- | --- | --- | --- | --- |
| Please rate the appropriateness of each item of the scales below on a 4-point Likert scale (1 = Not at Appropriate to 4 = Highly Appropriate) | Expert 1 | Expert 2 | Expert 3 | Expert 4 | Number of experts who scored 3 or 4 on the item | Item I-CVI |
| 1. I get back out there | X | X | X | X | **4** | **1.00** |
| 1. I still work to accomplish my goals | X | X | X | X | **4** | **1.00** |
| 1. I push through it | X | X | X | X | **4** | **1.00** |
| 1. I try to continue working | X | X | X | X | **4** | **1.00** |
| 1. I like to stay active | X | X | X | X | **4** | **1.00** |
| 1. I keep a positive attitude | X | X | X | X | **4** | **1.00** |
| 1. It doesn’t affect my happiness | X | X | X | X | **4** | **1.00** |
| 1. I still find joy in my life | X | X | X | X | **4** | **1.00** |
| 1. I keep a hopeful attitude | X | X | X | X | **4** | **1.00** |
| 1. I don’t let it get me down | X | X | X | X | **4** | **1.00** |
| 1. I avoid negative thoughts | X | X | ___ | X | **3** | **0.75** |
| 1. I try to stay relaxed | X | X | ___ | X | **3** | **0.75** |
| S-CVI/Ave | **0.958** | | | | | |
